# ASSOCIATION BETWEEN COUNTY TEMPERATURE AND VISION IMPAIRMENT IN A NATIONALLY REPRESENTATIVE SAMPLE OF OLDER ADULTS

**DOI:** 10.1093/geroni/igad104.0354

**Published:** 2023-12-21

**Authors:** Esme Fuller-Thomson, ZhiDi Deng, Elysia G Fuller-Thomson

**Affiliations:** University of Toronto, Toronto, Ontario, Canada; University of Alberta, Edmonton, Alberta, Canada; University of Toronto, Toronto, Ontario, Canada

## Abstract

Several small studies have associated exposure to elevated average temperature with specific vision problems. However, no large-scale studies have examined the relationship between vision impairment and average area temperature in the general population. We conducted a cross-sectional analysis of a large nationally presentative sample of older adults to further explore this relationship through secondary analysis of the American Community Survey (ACS). Data from six consecutive years of the cross-sectional survey were analyzed (2012-2017). The subsample analyzed included community-dwelling and institutionalized older adults aged 65 and older in the coterminous US who lived in the same state in which they were born (n =1,707,333). The question on severe vision impairment was “Is this person blind or does he/she have serious difficulty seeing even when wearing glasses?”. Average annual temperature data from the National Oceanic and Atmospheric Administration was combined into a 100-year average and mapped to corresponding US Census Bureau’s public use microdata areas (PUMAs) from the ACS. Higher average temperature consistently correlated with increased odds of severe vision impairment across all cohorts (i.e. age, sex, race, income, and educational attainment cohorts). Among all participants, the odds of severe vision impairment increased by 9.2% for every 5°F (2.8°C) elevation in annual average temperature (OR 1.092; 95% CI 1.088-1.097). If the association is found to be causal, the predicted rise in global temperatures could impact the number of older Americans affected by severe vision impairment and the associated health and economic burden.

